# Third Ventricular Tumors: A Comprehensive Literature Review

**DOI:** 10.7759/cureus.3417

**Published:** 2018-10-05

**Authors:** Syed Ijlal Ahmed, Gohar Javed, Altaf Ali Laghari, Syeda Beenish Bareeqa, Kashif Aziz, Mehreen Khan, Syeda Sana Samar, Raja Azhar Humera, Alizay Rashid Khan, Muhammad Osama Farooqui, Amir Shahbaz

**Affiliations:** 1 Neurosurgery, Liaquat National Hospital and Medical College, Karachi, PAK; 2 Neurosurgery, The Aga Khan University, Karachi, PAK; 3 Oncology, Jinnah Medical and Dental College, Karachi, PAK; 4 Internal Medicine, Icahn School of Medicine at Mount Sinai/Queens Hospital Center, New York, USA; 5 Internal Medicine, The George Washington University School of Medicine and Health Sciences, Washington DC, USA; 6 Internal Medicine, Jinnah Sindh Medical University, Karachi , PAK; 7 Internal Medicine, Ziauddin College of Medicine, Karachi, PAK; 8 Internal Medicine, Dow University of Health Sciences (DUHS), Karachi, PAK

**Keywords:** third ventricle tumors, ependymomas, pineal tumor, meningiomas, craniopharyngiomas

## Abstract

Third ventricle tumors are uncommon and account for 0.6 - 0.9% of all the brain tumors. Tumors of the third ventricle are classified into primary tumors, such as colloid cysts, choroid plexus papillomas, and ependymomas, or secondary tumors, such as craniopharyngiomas, optic nerve gliomas, pineal tumors, and meningiomas. Third ventricular tumors are uncommon, and their treatment involves significant morbidity and mortality. The colloid cyst has a better surgical outcome and many approaches are available to achieve a complete cure. Choroid plexus papilloma is also a common tumor documented with its treatment majorly based on surgical resection. In addition to multiple treatment options for craniopharyngiomas, surgery is the most preferred treatment option. Ependymomas also have few treatment options, with surgical resection adopted as the first line of treatment.

## Introduction and background

Third ventricle tumors are uncommon and account for only 0.6 - 0.9% of all the brain tumors [1]. We can classify them into primary tumors (e.g., colloid cysts, choroid plexus papillomas, and ependymomas) or secondary tumors (e.g., craniopharyngiomas, optic nerve gliomas, pineal tumors, and meningiomas) [2]. Third ventricle tumors also divided into the lesions involving either the anterior, posterior, or whole of the third ventricle. The division between the anterior and posterior portion of the third ventricle is based upon an imaginary line connecting foramen of Monro and aqueduct. The most common intraventricular tumors in children are choroid plexus papillomas, ependymomas, teratomas, and germinomas. Ten percent of choroid plexus papillomas occur predominantly in children younger than five years of age [3]. Ependymomas are the third most common primary third ventricular tumor found in children [4]. They constitute about 8 - 15% of the neoplasms [5].

In pediatric cases, the location is intracranial while, in adults, it is spinal. Colloid cysts are benign intracranial tumors usually occurring in the anterior part of the third ventricle [6]. In the young, they are assumed to be more aggressive clinically and radiologically and may require immediate intervention [7]. They are the most common tumors detected in adults in the anterior part of the third ventricle [3]. Their treatment involves significant morbidity and mortality. 

The objective of this paper is to review the medical literature regarding the treatment and outcome of third ventricle tumors, including the presentation, treatment options, and post-treatment complications.

## Review

The literature databases searched for our review were Medline and Google Scholar. The type of articles selected was case reports, cohort studies, surgeon general’s report, retrospective studies, and follow-up patient databases. The data collected were divided into primary and secondary tumors of the third ventricle and further subdivided into its subtypes. Data for the management and complications was then compiled. The data regarding patients in different age groups were added, ranging from infants to old age. The first choice treatment option for third ventricle lesions with dilated ventricles was endoscopic management [1]. Among microsurgical approaches, the expanded transcallosal transforaminal approach was a more recently practiced and safe method of accessing the anterior and middle third ventricle. With this approach, the risk of damage to most of the vital structures, such as the fornix or the thalamus, was avoided. The location of the junction of the anterior septal and internal cerebral vein is essential. Preoperative magnetic resonance (MR) venography can identify the junction. Some areas remain inaccessible, such as the anterosuperior and posterosuperior regions of the third ventricle.

Colloid cysts

Colloid cysts are cysts containing gelatinous material and mostly located at the level of or anterior to the foramen of Monro in the anterior aspect of the third ventricle. They represent 0.5 - 1% of intracranial tumors [8]. They are derived from either primitive neuroepithelium or endoderm. Most patients remain asymptomatic for an extended period, whereas some can present with vertigo, paroxysmal headaches, diplopia, memory deficits, gait disturbance, nausea, vomiting, and weakness of the lower limbs. In extreme cases, sudden death may occur [9]. Clinical presentation may be non-specific and heterogeneous. Colloid cysts are commonly associated with the development of hydrocephalus. This combination has an associated 3.1% risk of rapid death [10]. Colloid cysts have been known to cause deterioration of consciousness due to acute hydrocephalus in patients, whereas, in others, the cysts were discovered incidentally during epileptic seizure treatment. A prompt diagnosis of colloid cysts should be made and treated to avoid neurological deterioration, herniation, and death. Colloid cysts are surgically curable. There are multiple approaches to the third ventricle. Some factors determine the most feasible approach, such as the size of the tumor, the surgeon's experience, and the desired outcome of the operation. The expanded transcallosal transforaminal approach is a comfortable and safe method [11]. An endoscopic excision is also a safe and minimally invasive technique [7]. Smaller retractor tubes have been used for complete resection of colloid cysts [12]. The endoscopic approach to colloid cysts is performed through the foramen of Monro and considered quite a safe technique for the total resection of these cysts [13]. There are multiple angles of visualization and illumination. An endoscopic approach may result in less discomfort, shorter hospital stays, and fewer postoperative complications [14]. A few postoperative complications have been observed, such as transient/permanent memory deficits, surgical wound infection, cerebrospinal fluid (CSF) leak, meningitis, temporary potomania, and intraventricular hemorrhage [15].

Choroid plexus papillomas (CPP)

Choroid plexus papilloma is a rare, benign neuroepithelial intraventricular tumor of the choroid plexus. It can cause increased cerebrospinal fluid production, leading to increased intracranial pressure and hydrocephalus. The tumor is neuroectodermal in origin and similar in structure to a normal choroid plexus. They most commonly affect young children under the age of five and account for 0.4 - 0.6% of all intracranial neoplasms. Signs of the tumor resulting from increased intracranial pressure are present in 91% of patients with vomiting, homonymous visual field defects, and headache being the most common symptoms. Other symptoms are ear ringing and dizziness. CPP is treated with surgery and rarely progresses to become malignant. If it becomes malignant, patients with CPP are more likely to experience recurrence and metastasis. Therefore, a gross total resection (GTR) with chemotherapy is essential in preventing recurrence and prolonging survival [16]. The use of chemotherapy remains limited for CPP because most of the tumors are benign. Surgical treatment has a better outcome with the chances of a cure increasing to almost 100% due to recent advances in imaging, surgical approaches, and quality of intensive care [17]. Irradiation, followed by subtotal resection, to treat a growing residual tumor in the case of CPP provides an even higher chance of success [18]. Safaee et al. reported the success rate of surgical intervention in CPP [19]. Out of 24 patients, 20 underwent GTR while four had a subtotal resection (STR). Only three patients showed recurrence at the interval of 1.6, 3.3, and 8.5 years. Due to the potential adverse neurological sequelae associated with radiation to the developing brain, recommendations discourage its use in children less than three years of age [18].

Where radiation is inadvisable, high-dose chemotherapy (HDCT) and autologous peripheral blood stem cell transplantation (aPBSCT) are essential potential adjuncts in the treatment of infants. Due to the hypervascularity of the tumor, complications may occur during surgery. Therefore, this makes GTR challenging to achieve. Excessive bleeding leads to increased perioperative mortality, especially in infants and young children. Koh et al. discussed various ways that could be attempted to overcome difficulties in obtaining GTR of CPP [20]. One method was preoperative embolization. This method, although useful, may be challenging when it comes to passing the catheter through the tortuous feeding vessel. Therefore, one could consider neoadjuvant chemotherapy with or without a biopsy as the alternative treatment. Finally, chemotherapy after an initial incomplete resection decreases the size and vascularity of tumors to facilitate GTR at the second-look surgery [20]. On magnetic resonance imaging (MRI), they appear as homogeneous or heterogeneous tumors with a cauliflower appearance. Papillomas are typically iso- or hypointense on T1- and T2-weighted imaging but may demonstrate a heterogeneous hyperintensity on T2-weighted imaging; they enhance after contrast injection unless the tumor is highly calcified [17, 21]. Computed tomography (CT) scan revealed an isodense lobulated lesion with homogeneous contrast enhancement [21].

Craniopharyngiomas

Craniopharyngiomas are brain tumors derived from the embryonic tissue of the pituitary gland. They are also known as Rathke’s pouch tumors, hypophyseal duct tumors, or adamantinomas. They occur most frequently in children and also in men and women during the fifth and sixth decades of life [22]. They are slow-growing benign tumors. Patients may present with a balance disorder, dry skin, fever, fatigue, headache, hypersomnia and lethargy, polydipsia, polyuria, vision loss, and endocrinological disturbances and can be diagnosed by a radiological study, such as an MRI. Craniopharyngiomas have a very typical appearance on an MRI scan – a well-defined mass of solid and cystic parts. As it is a benign tumor and not expected to spread elsewhere, no further scans are required unless indicated by particular symptoms. Controversy exists regarding the treatment of choice for craniopharyngiomas; radical surgery, subtotal resection combined with radiotherapy, or primary irradiation are the available options [23]. The frontolateral approach is considered safe and straightforward. It gives sufficient access to even large craniopharyngiomas and promotes their complete removal with low to moderate morbidity [24]. Radical surgery is considered to be the treatment of choice as these are benign tumors. Surgical management commonly involves various transcranial routes. The endoscopic, endonasal, extended transsphenoidal approach is a minimal-access technique for managing smaller tumors. Also, it has a low rate of CSF leak [25]. The ‘extended’ endonasal approach overcomes the limits of the transsphenoidal route to the sella, enabling treatment of suprasellar and retrosellar extensions of the tumor (Video 1) [26-27]. Gamma Knife surgery can be useful in cases of recurrent lesions [28]. Short-term side effects from radiotherapy may include nausea, fatigue, and mild skin reactions. With the above methods, there are significant and comparable chances of visual improvement.

**Video 1:** An endonasal, fully endoscopic, transplanum transtuberculum approach for a craniopharyngioma [27]

Ependymomas

Ependymomas arise from the ependyma and are also seen with neurofibromatosis type II. They are composed of cells with regular, round to oval nuclei. Ependymomas are a neuroepithelial glial cell tumor. Up to 30% of intracranial ependymomas arise from the lateral or third ventricles [29]. Ependymomas make up about 5% of adult intracranial gliomas and up to 10% of childhood tumors of the central nervous system. Their occurrence peaks at age five years and then again at age 35. Ependymomas are the fourth most common posterior fossa tumors in children and constitute approximately 8 - 15% of the neoplasms [5]. Signs and symptoms include a severe headache, visual loss (secondary to papilledema), vomiting, bilateral Babinski sign, drowsiness, gait change (rotation of feet when walking), constipation, back pain, and abnormal flexibility of the back. Among treatment options, surgical resection is the first line of treatment. Studies have shown that adjuvant radiotherapy can be beneficial, but the ideal volume of irradiation remains controversial. The use of chemotherapy remains uncertain, and little evidence presently supports its use. Surgical treatment of intraventricular tumors has a high operative risk and, therefore, is associated with difficulties in the gross total resection of these tumors [30]. The prognosis for pediatric ependymomas remains relatively poor as compared to other brain tumors. In a study with 43 patients, all less than 18 years of age, underwent a combination of surgical excision, chemotherapy, and radiotherapy, but 16 patients died due to their ependymomas and 27 patients survived [29]. In another study, 36 patients were treated for intraventricular ependymomas and subependymoma of which 19 patients achieved complete resection while the remaining 17 underwent either subtotal or partial resection [30]. Eight patients died after surgery due to postoperative complications. Five patients were severely disabled postoperatively. Intraventricular ependymomas remain a surgical challenge due to their high rate of incomplete tumor resection and permanent neurological complications linked to their removal. Incomplete tumor removal accompanied by radiotherapy helps in long-term progression-free survival in a few cases [30].

Management of tumors in general

Important vascular and neural structures surround the tumors of the third ventricle. It is essential to see which approach is most suitable for which tumor. Neuroendoscopic management is considered to be the first and foremost line of treatment in particular with posterior third ventricle lesions. It is a minimally invasive procedure but with a significant risk of complications, so an experienced surgeon should perform the procedure. The same is also true for pediatric cases. The complications were linked with the histopathological origin of the tumor rather than the experience of the surgeon [31].

On the other hand, a biopsy is a safe procedure. A few complications were noted after surgery, such as transient fever, nausea and vomiting, and transient double vision [1]. A neuroendoscopic biopsy is the only treatment option available for some tumors, in particular, non-resectable ones [32].

There are three broad categories – anterior, lateral, and posterior routes. The anterior routes include transforaminal, interforniceal, transchoroidal, and subchoroidal. The subtemporal route is the main lateral corridor to the third ventricle and recommended if the tumor is located lateral to the sella turcica or extends into the middle cranial fossa [33]. The expanded transcallosal transforaminal approach remains a safe and relatively secure method of gaining access to the third ventricle [11]. A transtubular access to the third ventricle is also practical. It enables blunt dissection of the corpus callosum which may minimize retraction injuries. Three-dimensional endoscopic visualization, coupled with a transparent plastic retractor, provides absolute and undeviating monitoring of the surgical corridor [34]. In the third ventricle's anterior portion, the endoscopic endonasal approach permits surgical maneuverability. The lamina terminalis and tuber cinereum are thought to be safe entry points for this approach [35]. Tumors leading to the blockage of the Sylvian aqueduct can cause obstructive hydrocephalus; this calls for a CSF diversion procedure, endoscopic third ventriculostomy, combined with an endoscopic biopsy. Posterior third ventricular tumors should be approached using a combination of a rigid-flexible endoscope [36].

## Conclusions

Of the four major tumors of the third ventricle discussed, colloid cysts are the most well-documented cases. They have a better surgical outcome with many approaches to achieving remission with safe techniques. Minor evidence regarding postoperative complications was found. CPP is also a common tumor documented with its treatment majorly based on surgical resection. Its success has increased to 100% with new advancements in imaging and surgical approaches and leads to a complete cure with minor postoperative complications. Craniopharyngiomas have multiple options for treatment with radical surgery being preferred the most, according to the documented data. Radiotherapy has some short-term side effects, including nausea, fatigue, and mild skin reactions. Lastly, ependymomas also have few treatment options, with surgical resection adopted as the first line of treatment. The prognosis for patients with ependymomas is relatively poor compared to other brain tumors with a substantial number of the patients dying of the disease and few severely disabled postoperatively.
